# Potential Zoonotic Transmission of *Giardia duodenalis* between Children and Calves in Bangladesh

**DOI:** 10.1155/2023/8224587

**Published:** 2023-02-21

**Authors:** Junqiang Li, Md Robiul Karim, S. H. M. Faruk Siddiki, Yuancai Chen, Ziyang Qin, Farzana Islam Rume, Longxian Zhang

**Affiliations:** ^1^College of Veterinary Medicine, Henan Agricultural University, Zhengzhou 450046, China; ^2^Department of Medicine, Faculty of Veterinary Medicine and Animal Science, Bangabandhu Sheikh Mujibur Rahman Agricultural University, Gazipur 1706, Bangladesh; ^3^Department of Microbiology and Public Health, Patuakhali Science and Technology University, Barishal 8210, Bangladesh

## Abstract

*Giardia duodenalis* is a zoonotic protozoan parasite that causes gastrointestinal illness in humans and livestock. We studied the genetic diversity of *G. duodenalis* in children and calves from Bangladesh to determine its zoonotic potential. Fecal samples collected from children (299) and calves (699) were screened with nested PCR with primers targeting the *ssu rRNA* gene for *G. duodenalis*. Positive samples were further multilocus genotyped using the *β*-giardin (*bg*), glutamate dehydrogenase (*gdh*), and triose phosphate isomerase (*tpi*) genes. The overall infection rate of *G. duodenalis* was 21.1% (63/299) in children and 5.7% (40/699) in calves. There were no significant differences in infection with *G. duodenalis* among age groups, sex, and study areas in children and calves. Multilocus genotyping (MLG) of human *G. duodenalis* identified zoonotic assemblages A (34.0%, 18/53) and B (50.9%, 27/53) and a so-called ruminant-specific assemblage E (11.3%, 6/53), as well as two mixed assemblages, B/D (1/53) and B/E (1/53). Assemblage E predominated in calves (82.3%, 28/34), followed by assemblages A (11.8%, 4/34) and B (5.9%, 2/34). Overall, zoonotic assemblages A, B, and E were found in 6.0% (18/299), 9.0% (27/299), and 2.0% (6/299) of the children's stool samples, respectively, and 0.6% (4/699), 0.3% (2/699), and 4.0% (28/699) of the calf fecal samples, respectively. Although there was a difference in the distribution of subassemblages in humans (mostly AII) and calves (mostly AI), the zoonotic assemblages A, B, and E present in both children and calves suggest the potential for zoonotic transmission of *G. duodenalis*. This molecular study highlights the fact that *G. duodenalis* infections were common in the study areas, with potential zoonotic transmission between children and calves, implying that cattle might play a role in *G. duodenalis* zoonotic transmission.

## 1. Introduction

Giardiasis is an important zoonotic disease caused by *Giardia duodenalis* and affects both human and veterinary health worldwide [1]. Annually, 184 million people are estimated to have symptomatic giardiasis, with the majority of infections occurring in developing countries; however, the number of subclinical infections is expected to be much higher [2]. Giardiasis is generally a self-limiting clinical illness characterized by watery diarrhea, abdominal cramps, bloating, weight loss, and nutritional malabsorption in humans [3]. However, the occurrence of asymptomatic infections is common in both humans and animals [4].

The *G. duodenalis* life cycle comprises rapidly multiplying trophozoites and environmentally hardy cysts, which are released in the feces and spread via the fecal-oral route [5]. While the cysts cannot be distinguished morphologically, molecular biological analysis revealed that *G. duodenalis* isolates are genotypically divided into eight genetic assemblages designated A through H, where each assemblage has a distinct host range [6]. These assemblages also exhibit genetic variation, with assemblage A containing the well-recognized subassemblages AI, AII, and AIII, and assemblage B containing subassemblages BIII and BIV, which are not supported by phylogenetic analysis [7–9]. Various subassemblages are also present in assemblage E [10].

Multilocus genotyping (MLG) is used to identify the subtypes of *G. duodenalis* assemblages by analyzing the *β*-giardin (*bg*), glutamate dehydrogenase (*gdh*), and triose phosphate isomerase (*tpi*) loci [11]. With the help of these molecular tools, it is possible to better assess the disease burden brought on by the parasite's zoonotic transmission [12]. However, these analyses can occasionally be inaccurate, making it necessary to genotype isolates with multiple markers. Using concatenated sequence data thus allows for a more reliable clustering of isolates [6].

Smallholder farms with 1–5 animals used primarily for milk production dominate cattle farming in Bangladesh. In these farms, there is always close contact between humans and animals. Poor hygiene and sanitation combined with frequent exposure to animals and their excrement facilitate the zoonotic transmission of diseases. *Giardia duodenalis* has previously been associated with diarrhea in children from Bangladesh [13, 14] and a high prevalence of giardiasis has recently been reported among humans [15]. Calves are considered to be a reservoir for zoonotic parasites, and the transmission of parasites from cattle to human handlers has been indicated in Bangladesh and India [16, 17]. The purpose of this study was to accomplish the molecular characterization of the assemblages of *G. duodenalis* in children and calves, to assess its zoonotic transmission between children and calves and its public health significance.

## 2. Materials and Methods

### 2.1. Study Areas and Populations

**Figure 1:**
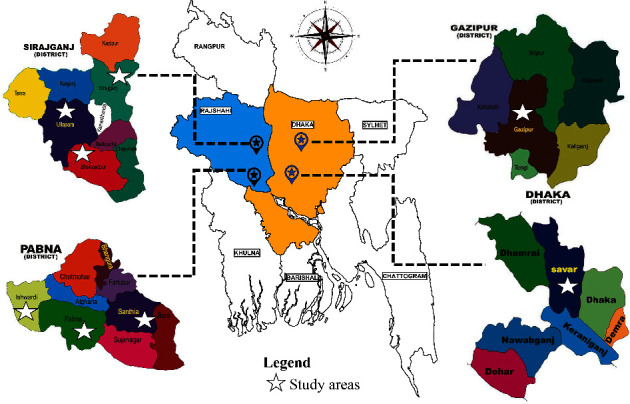
Sample collection areas in Bangladesh.

Eight sampling sites (subdistricts) located in four administrative regions (districts) were selected between July 2017 and June 2020, including the Sirajganj, Pabna, Gazipur, and Dhaka districts [18] (Figure 1). The Sirajganj and Paban districts are known for their milk production, with the majority of people being involved in cattle husbandry either directly or indirectly. The Gazipur and Dhaka districts are industrial areas. The study was largely conducted on smallholder dairy farms, except for one organized government dairy farm, the Central Cattle Breeding and Dairy Farm (CCBDF), which is located in the Savar subdistrict of the Dhaka district. The CCBDF was chosen to compare the findings on smallholder and organized dairy farms.

The target populations of this study were children and calves. Except for the CCBDF, the majority of samples came from children and calves living in the same household with direct or indirect contact with each other. The inclusion criteria were parents/guardians and farmers who consented to participate in the study. Children younger than 14 years old and calves younger than eight months old were sampled [18].

### 2.2. Survey and Fecal Sample Collection

The formula *n* = *Z*^2^*P*(1 − *P*)/*d*^2^ was used to determine the minimum sample sizes for this study, using prevalences of 10% and 30% for children and calves, respectively, with a desired precision of 5% at a 95% confidence level [19]. The minimum sample sizes were calculated as 139 for children and 323 for calves. In this study, 299 and 699 fresh fecal samples were collected from children and calves, respectively. Each participant provided one sample in a labeled zipper bag. Fresh calf feces were collected directly from the rectum and, in some cases, from the ground using disposable gloves. Polythene papers were provided for the children so that they could defecate on them before placing their samples in zipper bags. During sampling, additional data, such as age, sex, breed (for calves), and fecal consistency, were collected with the help of pretested questionnaires. Immediately after collection, samples were placed in an ice box and delivered to the laboratory. The samples were sieved, centrifuged, and stored at 4°C in 2.5% (w/v) potassium dichromate until DNA extraction.

### 2.3. DNA Extraction and PCR Amplification

The potassium dichromate was eliminated from the stored fecal samples by repeated washings with deionized water. The E.Z.N.A.^®^ Stool DNA kit (Omega Bio-tek Inc., Norcross, GA, USA) was used to extract total DNA from each fecal sample. Around 200 mg of the fecal sample was used for DNA extraction, as directed by the manufacturer's instructions. Finally, the obtained DNA was suspended in 200 *μ*L of elution buffer [20]. DNA samples were stored at 20°C until used for PCR. All samples were tested for *G. duodenalis* using a nested PCR amplification of the 290-bp fragment of the *ssu rRNA* gene (see [21] for reaction conditions, volumes, and primer information). The secondary PCR products were electrophoresed on a 1.5% agarose gel and examined using a transilluminator after being stained with ethidium bromide. Each round of PCR amplification comprised both a positive control (DNA of *G. duodenalis* assemblage B from a human) and a negative control (distilled water). All samples were screened in triplicate.

### 2.4. Multilocus Genotyping (MLG) of *G. duodenalis*

*Giardia duodenalis* samples positive by *ssu rRNA* PCR were subjected to MLG analysis, which included a nested PCR amplification of the *bg*, *gdh*, and *tpi* genes. We targeted nested PCRs to amplify fragments of the *bg*, *gdh*, and *tpi* genes under conditions previously described by Lalle et al. [22]; Appelbee et al. [23]; and Sulaiman et al. [24]; respectively, with some modifications [25, 26]. Each round of PCR amplification contained both positive and negative controls to ensure the accuracy of the results.

### 2.5. Nucleotide Sequencing and Analysis

Positive PCR amplicons were purified with Montage PCR filters (Millipore, Bedford, MA, USA). The ABI BigDye Terminator v. 3.1 cycle sequencing kit (Applied Biosystems, Foster City, CA, USA) and an ABI 3100 automated sequencer (Applied Biosystems) were used to sequence the amplicons in both directions using representative forward and reverse primers [22–24]. The forward and reverse nucleotide sequences and chromatograms were examined with EditSeq 5.0 (https://www.dnastar.com/) and Chromas 2.4 (https://technelysium.com.au/wp/chromas/), respectively. After using Chromas to ensure that there were no double peaks on the chromatogram, the sequences were aligned and analyzed with ClustalX (https://www.clustal.org/clustal2/). The assemblages and subtypes of *G. duodenalis* were identified by comparing consensus sequences to similar sequences in the GenBank database using the Basic Local Alignment Search Tool (BLAST) (https://www.ncbi.nlm.nih.gov/blast/).

### 2.6. Phylogenetic Analysis

Phylogenetic evolutionary analysis was conducted using the program Molecular Evolutionary Genetics Analysis (MEGA) version 6.0. In MEGA, sequences with no overlapping nucleotide bases were matched with representative sequences from the main *G. duodenalis* assemblages and subassemblages obtained from GenBank in the previous step. The evolutionary distances computed by the Kimura-2-parameter model were used to build neighbor-joining (NJ) trees. A bootstrap analysis with 1,000 replicates was used to evaluate the reliability of the trees and provide a consensus tree for illustration.

### 2.7. Nucleotide Sequence Accession Numbers and Statistical Analysis

The representative nucleotide sequences of this study were deposited in the National Center for Biotechnology Information (NCBI) GenBank database under the accession numbers: MK982540–MK982551, MN187867–MN187870, and MW055931 for the *bg* gene; MK982469–MK982483, MN187871–MN187872, and MW055932–MW055936 for the *gdh* gene; and MK982486–MK982497 for the *tpi* gene.

SPSS software was used for statistical analysis of the demographic data of the study participants. The potential relationships between *G. duodenalis* infections and sex, age groups, breeds of calves, and study regions were assessed using chi-squared tests, with calculated values being statistically significant when *p*  <  0.05.

## 3. Results

### 3.1. Demographic Features of Study Participants

Of the 299 children in this study, 178 (59.5%) were male, and 121 (40.5%) were female. The children were divided into two age groups, 0–5 years (175, 58.5%) and 5–14 years (124, 41.5%). The children ranged in age from 0.5 to 13 years, with a mean age of 4.46 years. Except for a few occurrences of soft stool noted during sample collection, the children appeared to be healthy. The 699 calves of this study fell into four breeds: 153 from a local breed (21.9%), 350 from the Holstein Friesian Cross (HFC) (50.1%), 178 from the Jersey Cross (JC) (25.5%), and 18 from the Brahman Cross (BrC) (2.6%). The calves belonged to three age groups, <1 month (66, 9.5%), 1–3 months (458, 65.5%), and >3 months (175, 25.0%). The calves ranged in age from 1 day to 8 months, with a mean age of 2.7 months. Of the calves, 352 (50.4%) were male and 347 (49.6%) were female. All calves were in good health, except for a few animals who had diarrhea.

### 3.2. Prevalence of *G. duodenalis* in Children and Calves

Of the stool samples from 299 children, 63 (21.1%) were positive for *G. duodenalis* infection by *ssu rRNA* PCR. The prevalence of *G. duodenalis* infections varied among the study sites, although the variation was not statistically significant (*p*  >  0.05) (Table 1). Similarly, the prevalence of *G. duodenalis* infection in children by sex and age group was statistically insignificant (Table 1). Meanwhile, *G. duodenalis* was detected in 40 (5.7%) of the 699 calf fecal samples (Table 1). Similarly, *G. duodenalis* infections in calves varied insignificantly across locations, sexes, age groups, and breed groups (*p*  >  0.05).

### 3.3. Sequence Analysis and Subtypes of *G. duodenalis* in Children

Among the 63 *G. duodenalis* positive children, 53 isolates were sequenced either for the *bg*, *gdh*, or *tpi* gene, and 24, 35, and 30 isolates were positive for the *bg*, *gdh*, and *tpi* genes, respectively (Table 2).

The *bg* gene sequences of the 24 human isolates revealed three assemblages: A (*n* = 9), B (*n* = 14), and D (*n* = 1). For assemblage A, subtypes A2 (MK982541, *n* = 4) and A3 (MK982540, *n* = 5) were identified, and these displayed 100% similarity with sequences obtained from human isolates in Egypt (MG736240) and Kenya (LC436576). For assemblage B, six different subtype sequences (*n* = 14) were identified, including three known (*n* = 11) and three novel sequences (*n* = 3). The three known sequence types, MK982542 (*n* = 9), MK982543 (*n* = 1), and MK982544 (*n* = 1), were identical to assemblage B isolates from humans in Ethiopia (KT948084), Brazil (KU504707), and Kenya (LC436567), respectively. The remaining three sequences from assemblage B (MK982545 (*n* = 1), MK982546 (*n* = 1), and MK982547 (*n* = 1)) represented novel isolates, which had five to seven nucleotide polymorphism substitutions compared to the reference subtype B3 (AY072726) (Supplementary Table 1). One isolate sequence was identified as assemblage D, which showed a 100% homology with a sequence previously reported from dogs in Italy (AY545647) and China (KY979500).

Among the 35 human isolates successfully amplified and sequenced from the *gdh* gene, assemblages A (*n* = 11), B (*n* = 17), and E (*n* = 7) were identified (Table 2). One sequence (MK982469, *n* = 11) was identified as assemblage A (subtype A2) which showed 100% similarity with an assemblage documented from children in China (MK962825) and humans in Iran (MH311029). There were 13 sequences from assemblage B identified from 17 isolates, including four known and nine novel sequences. The four known sequences, MK982470 (*n* = 3), MK982471 (*n* = 1), MK982472 (*n* = 2), and MK982473 (*n* = 1), were identical to those isolated from humans in Australia (EF685684), Ethiopia (KT948094), Egypt (MG746611), and Ethiopia (KT948095), respectively. Analysis of the nine novel sequences revealed that these isolates differed from the reference subtype B4 (EF507654) by four to 10 single nucleotide polymorphisms (Supplementary Table 2). The remaining seven isolates were identified as assemblage E, four of which (MK982483) belonged to a known subtype E3 (KY769099), and three belonged to a novel subtype. The novel sequence (MK982484) displayed 99.8% similarity to an isolate from Tan Sheep (MK645797) in China, with one nucleotide substitution at position 499 (G to A).

For the *tpi* gene, 30 human isolates were successfully amplified and sequenced and classified as assemblages A (*n* = 14) and B (*n* = 16). Three distinct sequence types were identified in the 14 assemblage A isolates, 10 isolates (MK982486) had 100% sequence similarity to subtype A2 obtained from humans in Iran (MH673818), Cuba (KY271712), Spain (KX469026), and Slovakia (KR105400). The sequences of the other three assemblage A isolates (MK982487) had a 100% similarity to subtype A2 reported in humans from Iran (MH673809) and monkeys from China (KJ888992). However, the sequence of the remaining isolate (MK982488) was 99.8% similar to human isolates from Iran (MH673818), with one substitution at nucleotide position 121 (A to T). The sequences of the 16 assemblage B isolates were assigned to nine subtypes, including four known and five novel subtypes. The four known sequence types MK982489 (*n* = 6), MK982490 (*n* = 2), MK982491 (*n* = 2), and MK982492 (*n* = 1) had 100% homology to human isolates from Iran (MH310999), Spain (KX468987), Croatia (JN587407), and Ethiopia (KT948104), respectively. The five novel sequence types (MK982493 to MK982497) were each detected from a single isolate, with two to seven nucleotide substitutions compared to the reference subtype B4 (AF069560) (Supplementary Table 3).

Overall, zoonotic assemblages A, B, and E were found in 6.0% (18/299), 9.0% (27/299), and 2.0% (6/299) of the children's stool samples, respectively.

### 3.4. Sequence Analysis and Subtypes of *G. duodenalis* in Calves

Among the 40 *G. duodenalis* positive calves, 34 isolates were sequenced either for the *bg*, *gdh*, or *tpi* gene, and 15, 26, and 0 isolates were positive for the *bg*, *gdh*, and *tpi* genes, respectively (Table 3).

The *bg* gene sequence analysis of the 15 isolates revealed three assemblages: A (*n* = 1), B (*n* = 2), and E (*n* = 12). The sequence of the zoonotic assemblage A (subtype A1) (MN187867) found in a calf was identical to the sequence found in cattle in China (KT698972) and the United States (MT713315), as well as humans in Sweden (GQ329671), sheep in China (MN833262), dogs in Japan (LC437420), deer in China (MF497409), and chipmunks in China (MF671918). Two different sequences of zoonotic assemblage B belonged to two novel subtypes (MK982548 and MN187868) (Table 3). The sequences of 12 assemblage E isolates comprised four known sequences: sequence MK982550 (*n* = 6) was identical to an Austrian calf isolate (MK202954); sequence MK982551 (*n* = 1) to a Spanish lamb isolate (EU726987); sequence MW055931 (*n* = 4) to a Chinese dairy cattle isolate (MF671887); and sequence MN187869 (*n* = 1) to a Chinese tan sheep isolate (MK610389).

Among the 26 isolates sequenced at the *gdh* gene, four were identified as assemblage A, and the remaining 22 were identified as assemblage E. The assemblage A isolates, MN187871 (*n* = 2), MW055935 (*n* = 1), and MW055936 (*n* = 1), were identical to an Ethiopian calf isolate (KT922255), a Brazilian human isolate (EF507676), and an Iranian human isolate (MH311029), respectively. Sequence analysis of the 22 assemblage E isolates revealed five subtypes, including two known and three novel subtypes. The known sequence MW055932 (*n* = 2) was identical to a Chinese cattle isolate (KY769099), and MK982485 (*n* = 7) was identical to an Australian human isolate (KY655475) and a Chinese dairy cattle isolate (MN602084). The novel subtype sequences were identified in one, eight, and four isolates, respectively (Table 3).

The known zoonotic assemblages A, B, and E were observed in 0.6% (4/699), 0.3% (2/699), and 4.0% (28/699) of the calf fecal samples, respectively.

### 3.5. Multilocus Genotyping of *G. duodenalis* in Children and Calves

Multilocus genotyping of *G. duodenalis* detected from children and calves were accomplished using the *bg*, *gdh,* and *tpi* genes. Of the 53 *G. duodenalis-*positive cases in children, 24, 35, and 30 were positive for the *bg*, *gdh*, and *tpi*, respectively. Among the 34 *G. duodenalis* positive isolates from calves, 15 and 26 isolates were successfully amplified and sequenced for the *bg* and *gdh* genes, respectively. As mentioned previously, the *tpi* gene was not amplified from any of the *G. duodenalis* positive calf fecal samples. However, it was amplified in 47.6% (30/63) of the human isolates.

**Figure 2:**
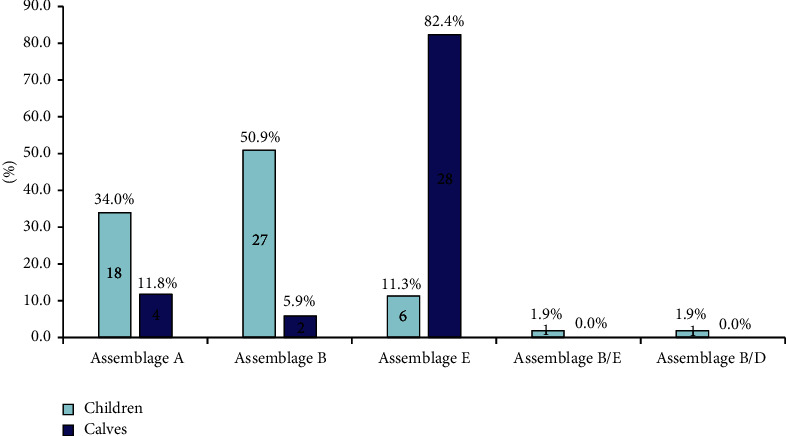
Distribution of *G. duodenalis* assemblages identified from children and calves.

DNA sequence analysis of the 53 human isolates of *G. duodenalis* showed that 50.9% (27/53) of the isolates belonged to assemblage B, 34.0% (18/53) to assemblage A, 11.3% (6/53) to assemblage E, 1.9% (1/53) to assemblage B/E, and 1.9% (1/53) to assemblage B/D. The *bg* and *gdh* gene sequences from the 34 isolates obtained from calves revealed the livestock-specific assemblage E and zoonotic assemblages A and B. *Giardia duodenalis* assemblage E was found in the majority of the calf isolates (82.3%, 28/34), while assemblages A and B were only found in 11.8% (4/34) and 5.9% (2/34) of the isolates, respectively. The assemblages of *G. duodenalis* identified from children and calves are shown in Figure 2.

Multiple alignment analysis of representative sequences obtained from assemblages A, B, D, and E revealed distinct sequence differences among the assemblages. A total of eight isolates were simultaneously amplified at these three loci, and subsequent analysis revealed six MLGs (MLG1 to MLG6) of *G. duodenalis* in children (Table 2).

### 3.6. Phylogenetic Analysis

Phylogenetic analysis of *G. duodenalis* isolates was performed using sequences from the *bg*, *gdh*, and *tpi* genes as well as tandem sequences of the *bg*, *gdh*, and *tpi* genes to clarify the evolutionary relationship between assemblage isolates of *G. duodenalis.*

**Figure 3:**
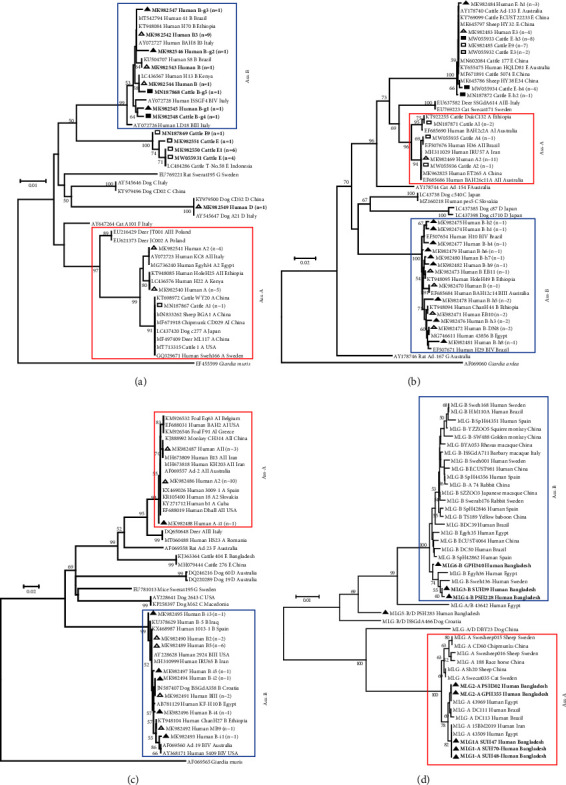
Phylogenetic relationships of *G. duodenalis* isolates as determined by the MEGA program using the sequences of the *bg* (a), *gdh* (b), and *tpi* (c) genes and tandem sequences of the *bg*, *gdh*, and *tpi* genes (d). Bootstrap values greater than 50 percent are shown on nodes. The neighbor joining (NJ) trees were constructed based on the evolutionary distances calculated by the Kimura-2-parameter model. The reliability of the trees was assessed using bootstrap analysis with 1,000 replicates. The children and calf isolates are indicated by triangle and rectangle shapes, respectively. The known and novel subtypes are indicated by hollow and filled shapes, respectively.

For the *bg* gene sequences, two known assemblage A sequences from children and one known assemblage A sequence from calves were subclustered near the subassemblages AII and AI, respectively. Three known and three novel assemblage B sequences from children, as well as two novel assemblage B sequences from calves, were clustered with assemblage B. One known assemblage D sequence from children and four known assemblage E sequences from calves were clustered with assemblages D and E, respectively (Figure 3(a)).

For the *gdh* gene sequences, the known assemblage A sequences, one from children and three from calves, were grouped with the assemblage A clade. There were 13 sequences from assemblage B identified from children, including three known and 10 novel sequences that were clustered into assemblage B. In the case of assemblage E, the child isolates belonged to two sequence types: one known and one novel, whereas the calf isolates belonged to two known and three novel sequences, all of which were grouped together (Figure 3(b)).

For the *tpi* gene sequences, two known and one novel assemblage A sequences from children were clustered with assemblage A. Meanwhile, four known assemblage B sequences and five novel types were clustered with assemblage B (Figure 3(c)). For the tandem sequences of these three genes, the isolates were clustered with assemblage A and assemblage B. The mixed assemblage isolates were clustered between them (Figure 3(d)).

## 4. Discussion

This study investigated the prevalence, genetic diversity, and zoonotic potential of *G. duodenalis* in children and calves from Bangladesh. A total of 21.1% (63/299) of the children were infected with *G. duodenalis*. Fewer infections of *G. duodenalis* were reported in children from Egypt (11.3%, 66/585) [27], Iran (7.06%, 20/283) [28], and the Netherlands (4.5%, 226/5015) [29]. A very low infection rate (1.56%, 67/4303) was reported among hospitalized children in Turkey [30], and China (0.61%, 14/2284) [31]. However, an infection rate similar to the results of this study was reported in school children (19.3%, 54/280) from Ethiopia [32]. On the other hand, higher infections were also documented in children with malignancy (68.5%, 37/54) in Bangladesh [15].

For calves, the overall prevalence of *G. duodenalis* infection was 5.7% (40/699). *Giardia duodenalis* infection rates in calves were much higher in Canada (42.0%, 60/143) [33], the United States (33.5%, 270/819) [34], Algeria (27.5%, 28/102) [35], and Austria (27.1%, 48/177) [36]. Higher infection rates of *G. duodenalis* were also reported from calves in Egypt (13.3%, 33/248) [37], Korea (12.7%, 40/315) [38], and Brazil (7.5%, 15/200) [39], while lower infection rates were reported in China (2.1%, 29/1366 and 2.2% 31/1440) [40, 41]. However, interestingly, a similar infection rate was reported in native Korean calves (5.6%, 44/792) [42]. In this study, *G. duodenalis* infection rates in children and calves varied insignificantly across age groups, sexes, and study areas. Many factors, such as host immune status, diet and feeding habits, sanitary conditions, management practices, and climatic conditions can influence differences in *G. duodenalis* prevalence across studies [43].

The genotypes of *G. duodenalis* in humans were mainly from assemblages A and B [1]. Many studies have confirmed this, with assemblages A and B being frequently reported in children in Egypt [36], Iran [44], Turkey [30], and China [31]. Occasionally, assemblages C, D, E, and F were documented in humans [45]. In the present study, assemblages A, B, and E, and B/D and B/E mixes were identified in children from Bangladesh.

In calves, *G. duodenalis* isolates mainly belonged to assemblage E, which is the predominant genotype found in cattle, sheep, and pigs [46, 47]. Several recent studies have confirmed the common occurrence of assemblage E isolates in calves from China [48], the United States [34], Austria [36], and Egypt [37]. The zoonotic assemblages A and B were found in 11.8% and 5.9% of the calf isolates from this study, respectively. However, assemblage A is likely more common in cattle than previously thought [6], as it has been frequently reported in calves in many countries, including Brazil [39], China [48], Korea [38], the United States [34], and Egypt [37]. In some studies, such as those conducted in China [40] and Canada [33], assemblage B was also frequently identified in calves.

Multilocus sequence analyses of the *bg*, *gdh*, and* tpi* genes have been used in subtyping assemblages A, B, and E [6]. Multilocus genotyping (MLG) of human isolates of *G. duodenalis* revealed the presence of zoonotic assemblages A (34.0%) and B (50.9%), and the so-called ruminant-specific assemblage E (11.3%) in children. However, the zoonotic potential of assemblage E has also been reported [49]. In the present study, the analyses of bovine isolates of *G. duodenalis* revealed the presence of cattle-specific assemblage E (82.3%), and zoonotic assemblages A (11.8%) and B (5.9%) in the isolates. More subtypes were generated at each of the three main genotyping loci in assemblage B than in assemblage A, making it more polymorphic than assemblage A [6]. The present study also uncovered a greater genetic variability in assemblage B compared with assemblages E, A, and D.

Previous studies showed that the infections of *G. duodenalis* detected among children were associated with contact with cattle in Australia, Ethiopia, and Ghana [50–52]. Calves may pose a risk for zoonotic transmission of *G. duodenalis* from cattle to humans [53]. The finding of zoonotic assemblages A (0.6%), B (0.3%), and E (4.0%) in calves in the present study further indicates the possibility of zoonotic human infections. Calves should thus be taken into consideration as an important reservoir for human giardiasis. This study also reported the so-called ruminant-specific *G. duodenalis* assemblage E in humans for the first time in Bangladesh. This assemblage was previously documented in humans from Brazil, Canada, China, and Egypt [45]. This observation suggests the cross-species transmission and zoonotic potential of assemblage E. Finally, the simultaneous identification of assemblages A, B, and E in both children and calves suggested the possibility of zoonotic transmission of *G. duodenalis* between humans and cattle.

## 5. Conclusions

*G. duodenalis* infections were widespread in children and calves from study areas in Bangladesh. Multilocus genotyping revealed notable genetic diversity among the *G. duodenalis* isolates from both children and calves. The presence of zoonotic assemblages A, B, and E in both children and calves suggests the possibility of zoonotic transmission of *G. duodenalis* between humans and cattle. This molecular characterization of the pathogen indicated that calves may play an important role in the zoonotic transmission of giardiasis in these study areas.

## Figures and Tables

**Table 1:** *Giardia duodenalis* infections in children and calves.

Parameters	No. of samples examined	No. of samples positive (%)	*χ* ^2^	*p* values
*Children* (*n* = 299)				
*Location*				
Sirajganj	140	32 (22.9)	2.291	0.318
Pabna	85	20 (23.5)		
Gazipur	74	11 (14.9)		
*Sex*				
Male	178	37 (20.8)	0.000	0.999
Female	121	26 (21.5)		
*Age group*				
<5 years	175	31 (17.7)	2.392	0.122
5–14 years	124	32 (25.8)		
*Calves* (*n* = 699)				
*Location*				
Sirajganj	213	17 (8.0)	3.799	0.284
Pabna	344	17 (4.9)		
Gazipur	52	1 (1.9)		
CCBDF (savar)	90	5 (5.6)		
*Sex*				
Male	352	19 (5.4)	0.044	0.834
Female	347	21 (6.1)		
*Age group*				
<1 month	66	4 (6.1)	0.016	0.992
1–3 months	458	26 (5.7)		
>3 months	175	10 (5.7)		
*Breed group*				
Local	153	8 (5.2)	6.358	0.095
HFC	350	23 (6.6)		
JC	178	6 (3.4)		
BrC	18	3 (16.7)		

*Note. χ* ^2^ and *p* values compare the prevalence between study sites, sex, and age in children and calves. HFC, JC, and BrC indicate Holstein Friesian cross, Jersey cross, and Brahman cross, respectively.

**Table 2:** Assemblages, subtypes, and MLGs of *Giardia duodenalis* as determined by sequence analysis of the *bg*, *gdh*, and *tpi* genes in children.

Study sites	Isolates	*Children*		*Assemblages (subtypes)*			MLGs
		Sex	Age^#^	*bg*	*gdh*	*tpi*	
Sirajganj	SH04	F	1.2	B (B3)	**B** ^*∗*^	-	
	SH05	M	0.8	A (A3)	-	-	
	SH06	M	1.5	-	E (E3)	-	
	SH14	M	12	-	**E** ^*∗*^		
	SH19	M	5	-	E (E3)	-	
	SH22	F	4.5	B (B3)	B (EB10)	-	
	SH26	M	7	-	**E** ^*∗*^	-	
	SH28	F	7	B (B3)	B (DN8)	-	
	SH43	M	13	-	E (E3)	-	
	SH46	F	5	A (A3)	A (A2)	-	
	SH53	F	7	B	E (E3)	-	
	SH56	F	2	B (B3)	B (EB11)	-	
	SH58	M	1.5	-	**E** ^*∗*^	-	
	SH72	M	2	B (B3)	**B** ^*∗*^	-	
	SH76	M	2	**B** ^*∗*^	-	-	
	SUH39	M	5	B (B3)	**B** ^*∗*^	B (B5)	MLG3
	SUH40	M	7	-	-	B (B5)	
	SUH44	M	2	-	**B** ^*∗*^	B (B2)	
	SUH45	M	10	-	A (A2)	A (A2)	
	SUH47	F	4	A (A3)	A (A2)	A (A2)	MLG1
	SUH48	F	8	A (A3)	A (A2)	A (A2)	MLG1
	SUH50	M	10	-	**B** ^*∗*^	B (B2)	
	SUH58	F	4	-	A (A2)	A (A2)	
	SUH70	M	7	A (A3)	A (A2)	A (A2)	MLG1
	SNH107	F	0.5	-	-	A (A2)	

Pabna	PH05	F	6	B (B3)	**B** ^*∗*^	-	
	PH44	M	4	**B** ^*∗*^	-	-	
	PH50	F	3	B (B3)	-	-	
	PH64	M	1	**B** ^*∗*^	-	-	
	PSH202	M	7	-	A (A2)	A (A2)	
	PSH217	F	4.5	-	A (A2)	A (A2)	
	PSH218	M	4.5	-	B	B (B3)	
	PSH225	F	7	-	-	**B** ^*∗*^	
	PSH228	M	8	B (B3)	**B** ^*∗*^	B (B5)	MLG4
	PSH245	F	9	-	-	A (A2)	
	PSH250	M	9	-	-	B (B5)	
	PSH260	F	7	-	-	B (B5)	
	PSH282	F	0.8	-	-	B (B5)	
	PSH283	M	4	D	**B** ^*∗*^	**B** ^*∗*^	MLG5
	PSH297	F	3	-	A (A2)	-	
	PSH302	M	4	A (A2)	A (A2)	A (A2)	MLG2
	PSH303	M	4	-	-	**A** ^*∗*^	
	PSH306	M	8	-	-	A (A2)	
	PSH307	M	7	-	B	B (MB9)	

Gazipur	GH02	F	6	-	**B** ^*∗*^	-	
	GH11	M	3	-	**B** ^*∗*^	-	
	GPH337	M	9	-	-	**B** ^*∗*^	
	GPH340	F	8	B	B	B (B3)	MLG6
	GPH304	F	1	A (A2)	-	-	
	GPH336	M	5	-	B (DN8)	**B** ^*∗*^	
	GPH355	F	9	A (A2)	A (A2)	A (A2)	MLG2
	GPH362	F	3	-	-	**B** ^*∗*^	
	GPH347	M	0.6	A (A2)	-	A (A2)	

*Note.* Asterisks (^*∗*^) indicate novel genotypes; hyphens (-) indicate PCR-negative results; hash (**#**) indicates age in year; M and F indicate male and female, respectively.

**Table 3:** Assemblages and subtypes of *Giardia duodenalis* as determined by sequence analysis of the *bg* and *gdh* genes in calves.

Study sites	Isolates	*Calf*			*Assemblages (subtypes)*	
		Sex	Age^#^	Breed	*bg*	*gdh*
Sirajganj	SC06	M	1	HFC	E (Ov230)	-
	SC17	M	5	HFC	-	E (E8)
	SC25	F	8	HFC	E (E1)	-
	SC60	M	3	HFC	E (E1)	E (E9)
	SC81	M	4	BrC	E (E9)	E (E3)
	SC119	M	4.5	Local	**B** ^*∗*^	-
	SUC7	F	2	HFC	-	**E** ^*∗*^
	SUC15	F	2	HFC	E (E1)	**E** ^*∗*^
	SUC22	F	1	HFC	-	**E** ^*∗*^
	SUC34	M	1	HFC	E (E1)	**E** ^*∗*^
	SUC78	M	2	JC	-	**E** ^*∗*^
	SUC99	F	3	HFC	-	**E** ^*∗*^

Pabna	PC09	M	0.4	Local	-	E (E9)
	PC98	F	3.5	HFC	-	**E** ^*∗*^
	SPC66	F	6	HFC	E (E1)	E (E8)
	SPC06	M	5	JC	**B** ^*∗*^	-
	SPC48	F	0.7	HFC	-	E (E8)
	SPC52	M	1	JC	A (A1)	A (A1)
	SPC55	M	7	HFC	-	E (E3)
	SPC73	M	0.7	HFC	-	E (E8)
	PIC36	M	2	JC	E	-
	PIC54	F	2.1	JC	E	-
	PIC78	M	0.7	Local	E	-
	PIC107	F	1	HFC	-	A1
	PIC125	F	1.7	Local	E	-
	PIC140	F	2	Local	-	**E** ^*∗*^
	PIC164	F	1	Local	-	**E** ^*∗*^
	PIC191	M	2	Local	-	**E** ^*∗*^
	PIC203	F	1.3	Local	-	**E** ^*∗*^

Gazipur	GC38	F	3	HFC	-	E (E8)
CCBDF (savar)	SD407	M	1.5	BrC	E (E1)	**E** ^*∗*^
	SD6653	F	2.8	HFC	-	A (A4)
	SD6701	F	1.4	JC	-	A (A2)
	SD6714	F	1.4	HFC	-	**E** ^*∗*^

*Note.* Asterisks (^*∗*^) indicate novel genotypes; hyphens (-) indicate PCR-negative results; hash (**#**) indicates age in month; M and F indicate male and female, respectively.

## Data Availability

Both this manuscript and the supplementary materials contain publicly available data that back up the study's findings.

## Supplementary Materials

Supplementary Materials The supplementary tables demonstrate the genetic variations within subtypes of *G. duodenalis* assemblages A and B at the *bg* (Supplementary Table 1), *gdh* (Supplementary Table 2), and *tpi* (Supplementary Table 3) genes in children.
