# Supplementary material for: Potential Zoonotic Transmission of Giardia duodenalis between Children and Calves in Bangladesh
Source: Transbound Emerg Dis. 2023 Feb 21;2023:8224587. doi: 10.1155/2023/8224587 (PMC12017152; doi:10.1155/2023/8224587)
Supplement: Supplementary Materials — The supplementary tables demonstrate the genetic variations within subtypes of G. duodenalis assemblages A and B at the bg (Supplementary Table 1), gdh (Supplementary Table 2), and tpi (Supplementary Table 3) genes in children. [file 8224587.f1.zip › Supplementary Table 1.docx]

Supplementary Table 1. Genetic variations within subtypes of *G. duodenalis* assemblages A and B at the *bg* gene in children

| **Assemblages/**  **subtypes** | **GenBank accession no.** | **No. of isolates** | **Nucleotide at positions** | | | | | | | | | | | | | | |
| --- | --- | --- | --- | --- | --- | --- | --- | --- | --- | --- | --- | --- | --- | --- | --- | --- | --- |
|  |  |  | **90** | **91** | **92** | **120** | **183** | **228** | **240** | **245** | **263** | **276** | **289** | **309** | **312** | **346** | **354** |
| **Ass-A** |  |  |  |  |  |  |  |  |  |  |  |  |  |  |  |  |  |
| A2 (Ref.) | KT948085 |  | A | C | A | G | G | C | T | A | A | A | C | C | C | T | C |
| A3 | MK982540 | 5 | * | * | * | * | * | * | * | * | * | * | * | * | * | * | * |
| A2 | MK982541 | 4 | * | * | * | * | * | * | * | * | * | * | * | * | * | C | T |
| **Ass-B** |  |  |  |  |  |  |  |  |  |  |  |  |  |  |  |  |  |
| B3 (Ref.) | AY072726 |  | G | G | A | C | A | G | T | A | T | C | G | C | C | A | G |
| B3 | MK982542 | 9 | A | A | C | * | * | A | * | * | * | * | * | * | T | * | * |
| B | MK982543 | 1 | * | * | * | * | * | A | C | * | * | * | * | * | T | * | * |
| B | MK982544 | 1 | * | * | G | * | * | A | * | * | * | * | * | T | T | * | * |
| B-g1 | MK982545 | 1 | * | * | * | T | G | A | * | * | * | * | A | * | T | * | * |
| B-g2 | MK982546 | 1 | A | A | C | * | * | A | * | * | C | T | * | * | T | * | * |
| B-g3 | MK982547 | 1 | A | A | C | * | * | A | * | G | * | * | * | * | T | * | * |

**Key:** Asterisks (*) indicate nucleotide identity with the reference sequence. Nucleotide positions are numbered according to the reference (ref.) assemblage partial sequences with the first nucleotide as position 23 for assemblage A (subtype A2, Ref.) sequence (GenBank accession number KT948085) and position 1 for assemblage B (subtype B3, Ref.) sequence (AY072726). Here, B-g1 to B-g3 are novel subtypes identified in this study.
