# Supplementary material for: Potential Zoonotic Transmission of Giardia duodenalis between Children and Calves in Bangladesh
Source: Transbound Emerg Dis. 2023 Feb 21;2023:8224587. doi: 10.1155/2023/8224587 (PMC12017152; doi:10.1155/2023/8224587)
Supplement: Supplementary Materials — The supplementary tables demonstrate the genetic variations within subtypes of G. duodenalis assemblages A and B at the bg (Supplementary Table 1), gdh (Supplementary Table 2), and tpi (Supplementary Table 3) genes in children. [file 8224587.f1.zip › Supplementary Table 2.docx]

Supplementary Table 2. Genetic variations within subtypes of *G. duodenalis* assemblage B at the *gdh* gene in children

| **Subtypes/**  **isolates** | **GenBank Accession no.** | **No. of isolates** | **Nucleotide at positions** | | | | | | | | | | | | | | | | | | | | | |
| --- | --- | --- | --- | --- | --- | --- | --- | --- | --- | --- | --- | --- | --- | --- | --- | --- | --- | --- | --- | --- | --- | --- | --- | --- |
|  |  |  | **288** | **354** | **375** | **381** | **396** | **405** | **441** | **447** | **504** | **534** | **558** | **642** | **650** | **660** | **669** | **702** | **726** | **753** | **756** | **768** | **782** | **807** |
| B4 (Ref.) | EF507654 |  | C | T | C | C | C | C | C | G | C | C | C | T | A | A | C | C | T | G | G | C | G | C |
| B (BAH12c14) | MK982470 | 1 | T | C | * | * | * | * | * | * | * | T | * | G | * | * | * | * | * | * | * | T | * | A |
| B (EB10) | MK982471 | 2 | T | C | * | * | * | * | * | A | * | * | T | * | * | G | * | * | * | * | A | * | * | A |
| B (DN8) | MK982472 | 2 | T | C | T | * | * | * | * | A | * | * | T | * | * | G | * | * | * | * | * | * | * | A |
| B (EB11) | MK982473 | 1 | T | C | * | * | * | * | * | * | * | * | * | * | * | * | * | * | * | * | A | * | * | A |
| B-h1 | MK982474 | 1 | T | * | * | * | * | * | * | * | * | * | * | * | * | * | T | T | * | * | * | * | * | A |
| B-h2 | MK982475 | 1 | T | * | * | T | * | * | * | * | * | * | * | * | G | * | T | * | * | * | * | * | * | A |
| B-h3 | MK982476 | 2 | T | C | T | * | * | * | T | A | * | * | * | * | * | G | * | * | * | * | A | * | * | A |
| B-h4 | MK982477 | 1 | T | * | * | T | * | * | * | * | * | * | T | * | * | G | T | * | * | * | * | * | * | A |
| B-h5 | MK982478 | 2 | T | C | T | * | * | T | * | A | T | * | T | G | * | * | * | * | * | * | * | * | * | A |
| B-h6 | MK982479 | 1 | T | C | * | * | * | * | * | * | * | * | T | * | * | * | * | * | * | * | * | * | * | A |
| B-h7 | MK982480 | 1 | T | C | * | * | * | * | * | * | * | * | * | G | * | * | * | * | * | * | A | * | * | A |
| B-h8 | MK982481 | 1 | T | C | T | * | T | * | * | * | * | * | T | * | * | G | * | * | C | * | * | T | A | A |
| B-h9 | MK982482 | 1 | T | C | * | * | * | * | * | * | * | * | T | G | * | * | * | * | * | A | A | * | * | A |

**Key:** Asterisks (*) indicate nucleotide identity with the reference sequence. Nucleotide positions are numbered according to the reference (ref.) assemblage B (subtype B4) partial sequence (GenBank accession number EF507654), with the first nucleotide as position 1. Here, B-h1 to B-h9 are novel subtypes identified in this study.
