# Supplementary material for: Potential Zoonotic Transmission of Giardia duodenalis between Children and Calves in Bangladesh
Source: Transbound Emerg Dis. 2023 Feb 21;2023:8224587. doi: 10.1155/2023/8224587 (PMC12017152; doi:10.1155/2023/8224587)
Supplement: Supplementary Materials — The supplementary tables demonstrate the genetic variations within subtypes of G. duodenalis assemblages A and B at the bg (Supplementary Table 1), gdh (Supplementary Table 2), and tpi (Supplementary Table 3) genes in children. [file 8224587.f1.zip › Supplementary Table 3.docx]

Supplementary Table 3. Genetic variations within subtypes of *G. duodenalis* assemblages A and B at the *tpi* gene in children

| **Assemblages/subtypes** | **GenBank accession no.** | **No. of isolates** | **Nucleotide at positions** | | | | | | | | | | | | | |
| --- | --- | --- | --- | --- | --- | --- | --- | --- | --- | --- | --- | --- | --- | --- | --- | --- |
|  |  |  | **13** | **65** | **68** | **76** | **121** | **136** | **139** | **142** | **184** | **244** | **388** | **403** | **419** | **478** |
| **Ass-A** |  |  |  |  |  |  |  |  |  |  |  |  |  |  |  |  |
| A2 (Ref.) | AF069557 |  | G | C | A | T | A | A | C | G | G | G | C | G | G | T |
| A2 | MK982486 | 10 | * | * | * | * | * | * | * | * | * | * | * | * | * | * |
| A2 | MK982487 | 3 | * | * | * | * | * | * | * | * | * | * | * | * | A | * |
| A-i1 | MK982488 | 1 | * | * | * | * | T | * | * | * | * | * | * | * | * | * |
| **Ass-B** |  |  |  |  |  |  |  |  |  |  |  |  |  |  |  |  |
| B4 (Ref.) | AF069560 |  | A | T | A | C | T | G | T | T | A | C | T | A | G | C |
| B5 | MK982489 | 6 | * | C | * | * | * | * | C | C | G | * | * | G | * | * |
| B2 | MK982490 | 2 | G | C | * | * | * | * | C | C | G | * | * | G | * | * |
| B3 | MK982491 | 2 | G | * | * | * | * | * | C | C | G | * | * | G | * | * |
| B (MB9) | MK982492 | 1 | * | * | * | * | * | * | * | C | G | * | * | G | * | * |
| B-i1 | MK982493 | 1 | * | * | G | * | * | * | * | * | * | * | * | G | * | * |
| B-i2 | MK982494 | 1 | G | * | * | * | * | * | C | C | G | T | * | G | * | * |
| B-i3 | MK982495 | 1 | G | C | * | * | * | * | C | C | G | * | C | G | * | T |
| B-i4 | MK982496 | 1 | G | * | * | T | * | A | C | C | G | * | * | G | * | * |
| B-i5 | MK982497 | 1 | * | C | * | * | * | * | C | C | * | * | * | G | * | * |

**Key:** Asterisks (*) indicate nucleotide identity with the reference sequence. Nucleotide positions are numbered according to the reference (ref.) assemblages A (subtype A2, GenBank accession number AF069557) and B (subtype B4, AF069560) partial sequences, with the first nucleotide as position 9. Here, B-i1 to B-i5 are novel subtypes identified in this study.
